# Correction: The evidence supporting AHA guidelines on adult cardiopulmonary resuscitation (CPR)

**DOI:** 10.1371/journal.pone.0326211

**Published:** 2025-06-10

**Authors:** Emma Junedahl, Peter Lundgren, Erik Andersson, Vibha Gupta, Truls Råmunddal, Aidin Rawshani, Gabriel Riva, Ida Arnetorp, Fredrik Hessulf, Johan Herlitz, Therese Djärv, Araz Rawshani

The authors are listed out of order. Please view the correct author order, affiliations, and citation here:

Emma Junedahl^1^, Peter Lundgren^1^, Erik Andersson^1^, Vibha Gupta^1^, Truls Råmunddal^1^, Aidin Rawshani^1^, Gabriel Riva^2^, Ida Arnetorp^1^, Fredrik Hessulf^3^, Johan Herlitz^3^, Therese Djärv^2^, Araz Rawshani^1,3^

**1** Department of Molecular and Clinical Medicine, Institute of Medicine University of Gothenburg, Gothenburg, Sweden, **2** Center for Resuscitation Science, Department of Science and Education KI SOS, Karolinska Institutet, Solna, Sweden, **3** The Swedish Cardiopulmonary Resuscitation Registry, Gothenburg, Sweden.
