# Supplementary material for: The evidence supporting AHA guidelines on adult cardiopulmonary resuscitation (CPR)
Source: PLoS One. 2024 Dec 17;19(12):e0309241. doi: 10.1371/journal.pone.0309241 (PMC11651587; doi:10.1371/journal.pone.0309241)
Supplement: S1 File — (PDF) [file pone.0309241.s002.pdf]

Heading	LOE	COR
Recommendations for Recognition of Cardiac Arres	1	C-LD
Recommendations for Recognition of Cardiac Arres	1	C-LD
Recommendations for Initiation of Resuscitation: Lay Rescuer (Untrained or Trained)	1	B-NR
Recommendations for Initiation of Resuscitation: Lay Rescuer (Untrained or Trained)	1	C-LD
Recommendations for Initiation of Resuscitation: Lay Rescuer (Untrained or Trained)	1	C-LD
Recommendations for Initiation of Resuscitation: Lay Rescuer (Untrained or Trained)	2a	C-LD
Recommendations for Initiation of Resuscitation: Lay Rescuer (Untrained or Trained)	1	B-NR
Recommendations for Initiation of Resuscitation: Lay Rescuer (Untrained or Trained)	1	C-LD
Recommendations for Initiation of Resuscitation: Lay Rescuer (Untrained or Trained)	1	C-LD
Recommendations for Initiation of Resuscitation: Lay Rescuer (Untrained or Trained)	2a	C-LD

**Recommdendations for Initiation of Resuscitation: Healthcare Provider**	1	C-LD
**Recommdendations for Initiation of Resuscitation: Healthcare Provider**	2a	C-LD
**Recommendations for Opening the Airway**	1	C-EO
**Recommendations for Opening the Airway**	1	C-EO
**Recommendations for Opening the Airway**	2b	C-EO
**Recommendations for Opening the Airway**	2a	C-EO
**Recommendations for Opening the Airway**	3: No Benefit	C-LD

**Recommendations for Opening the Airway After Head and Neck Trauma**	1	C-EO
**Recommendations for Opening the Airway After Head and Neck Trauma**	1	C-EO
**Recommendations for Opening the Airway After Head and Neck Trauma**	3: Harm	C-LD
**Recommendations for Positioning and Location for CPR**	1	C-LD
**Recommendations for Positioning and Location for CPR**	1	C-EO

**Recommendations for Positioning and Location for CPR**	2a	C-LD
**Recommendations for Positioning and Location for CPR**	2b	C-LD
**Recommendations for Compression Fraction and Pauses**	1	C-LD
**Recommendations for Compression Fraction and Pauses**	1	C-LD
**Recommendations for Compression Fraction and Pauses**	2a	B-R

**Recommendations for Compression Fraction and Pauses**	2a	B-R
**Recommendations for Compression Fraction and Pauses**	2a	C-LD
**Recommendations for Compression Fraction and Pauses**	2b	C-LD
**Recommendations for Compression Depth and Rate**	1	B-NR
**Recommendations for Compression Depth and Rate**	2a	B-NR
**Recommendations for Compression Depth and Rate**	2a	C-LD

**Recommendations for Compression Depth and Rate**	2b	C-EO
**Recommendations for CPR Feedback and Monitoring**	2b	B-R
**Recommendations for CPR Feedback and Monitoring**	2b	C-LD
**Recommendations for Ventilation During Cardiac Arrest: Special Situations**	2a	C-LD
**Recommendations for Ventilation During Cardiac Arrest: Special Situations**	2b	C-EO
**Recommendations for Fundamentals of Ventilation During Cardiac Arrest**	2a	C-LD

**Recommendations for Fundamentals of Ventilation During Cardiac Arrest**	2a	C-EO
**Recommendations for Fundamentals of Ventilation During Cardiac Arrest**	2b	C-EO
**Recommendations for Fundamentals of Ventilation During Cardiac Arrest**	3: Harm	C-LD
**Ventilation in Patients With Spontaneous Circulation (Respiratory Arrest)**	2b	C-LD
**Recommendations for Compression-to-Ventilation Ratio: ALS**	2a	B-R

**Recommendations for Compression-to-Ventilation Ratio: ALS**	2b	B-R
**Recommendations for Compression-to-Ventilation Ratio: ALS**	2b	C-LD
**Recommendations for Compression-to-Ventilation Ratio: ALS**	2b	C-LD
**Recommendations for Defibrillation Indication, Type, and Energy**	1	B-NR
**Recommendations for Defibrillation Indication, Type, and Energy**	2a	B-R

**Recommendations for Defibrillation Indication, Type, and Energy**	2a	B-NR
**Recommendations for Defibrillation Indication, Type, and Energy**	2a	C-LD
**Recommendations for Defibrillation Indication, Type, and Energy**	2b	B-R
**Recommendations for Defibrillation Indication, Type, and Energy**	2b	C-LD

**Pads for Defibrillation**	2a	C-LD
**Automatic- Versus Manual-Mode Defibrillation**	2b	C-LD
**Recommendations for CPR Before Defibrillation**	1	C-LD
**Recommendations for CPR Before Defibrillation**	2a	B-R
**Recommendations for CPR Before Defibrillation**	2a	C-LD

**Postshock Rhythm Check**	2b	C-LD
**Anticipatory Defibrillator Charging**	2b	C-EO
**Recommendations for Ancillary Defibrillator Technologies**	2b	C-LD
**Recommendations for Ancillary Defibrillator Technologies**	2b	C-LD
**Double Sequential Defibrillation**	2b	C-LD

Recommendations for Precordial Thump	2b	B-NR
Recommendations for Precordial Thump	3: No Benefit	C-LD
Electric Pacing	3: No Benefit	B-R
Fist/Percussion Pacing	2b	C-LD

**Cough CPR**	2b	C-LD
**Recommendations for Vascular Access in Cardiac Arrest Management**	2a	B-NR
**Recommendations for Vascular Access in Cardiac Arrest Management**	2b	B-NR
**Recommendations for Vascular Access in Cardiac Arrest Management**	2b	C-LD
**Recommendations for Vascular Access in Cardiac Arrest Management**	2b	C-LD
**Recommendations for Vasopressor Management in Cardiac Arrest**	1	B-R

**Recommendations for Vasopressor Management in Cardiac Arrest**	2a	B-R
**Recommendations for Vasopressor Management in Cardiac Arrest**	2a	C-LD
**Recommendations for Vasopressor Management in Cardiac Arrest**	2b	C-LD
**Recommendations for Vasopressor Management in Cardiac Arrest**	2b	C-LD
**Recommendations for Vasopressor Management in Cardiac Arrest**	3: No Benefit	B-R
**Recommendations for Nonvasopressor Medications**	2b	B-R
**Recommendations for Nonvasopressor Medications**	2b	C-LD

**Recommendations for Nonvasopressor Medications**	3: No Benefit	B-NR
**Recommendations for Nonvasopressor Medications**	3: No Benefit	B-R
**Recommendations for Nonvasopressor Medications**	3: No Benefit	B-R
**Recommendations for Adjuncts to CPR**	2b	C-LD
**Recommendations for Adjuncts to CPR**	2b	C-LD
**Recommendations for Adjuncts to CPR**	2b	C-LD
**Recommendations for Adjuncts to CPR**	2b	C-EO

**Recommendations for Adjuncts to CPR**	2b	C-EO
**Recommendations for Termination of Resuscitation**	1	B-NR
**Recommendations for Termination of Resuscitation**	2a	B-NR
**Recommendations for Termination of Resuscitation**	2a	B-NR

**Recommendations for Termination of Resuscitation**	2b	C-LD
**Recommendations for Termination of Resuscitation**	3: No Benefit	C-LD
**Recommendations for Termination of Resuscitation**	3: Harm	C-EO
**Advanced Airway Interventions During Cardiac Arrest**	2b	B-R
**Recommendations for Advanced Airway Placement Considerations**	1	B-NR

**Recommendations for Advanced Airway Placement Considerations**	1	C-LD
**Recommendations for Advanced Airway Placement Considerations**	1	C-LD
**Recommendations for Advanced Airway Placement Considerations**	1	C-EO
**Recommendations for Choice of Advanced Airway Device: Endotracheal Intubation Versus Supraglottic Airway**	2a	B-R

**Recommendations for Choice of Advanced Airway Device: Endotracheal Intubation Versus Supraglottic Airway**	2a	B-R
**Recommendations for Choice of Advanced Airway Device: Endotracheal Intubation Versus Supraglottic Airway**	2a	B-R
**Recommendations for Active Compression-Decompression CPR and Impedance Threshold Devices**	2b	B-NR
**Recommendations for Active Compression-Decompression CPR and Impedance Threshold Devices**	2b	C-LD

**Recommendations for Active Compression-Decompression CPR and Impedance Threshold Devices**	3: No Benefit	A
**Recommendations for Mechanical CPR Devices**	2b	C-LD
**Recommendations for Mechanical CPR Devices**	3: No Benefit	B-R
**Alternative CPR Techniques**	2b	B-NR

Extracorporeal CPR	2b	C-LD
Recommendations for Pharmacological Management of Hemodynamically Stable Wide-Complex Tachycardia	2b	B-NR
Recommendations for Pharmacological Management of Hemodynamically Stable Wide-Complex Tachycardia	2b	B-R
Recommendations for Pharmacological Management of Hemodynamically Stable Wide-Complex Tachycardia	3: Harm	B-NR

Recommendations for Pharmacological Management of Hemodynamically Stable Wide-Complex Tachycardia	3: Harm	C-LD
Electric Management of Hemodynamically Stable Wide-Complex Tachycardia	2a	C-LD
Electric Treatment of Polymorphic VT	1	B-NR
Pharmacological Treatment of Polymorphic VT Associated With a Long QT Interval (Torsades De Pointes)	2b	C-LD
Recommendations for Pharmacological Treatment of Polymorphic VT Not Associated With a Long QT Interval	2b	C-LD
Recommendations for Pharmacological Treatment of Polymorphic VT Not Associated With a Long QT Interval	3: No Benefit	C-LD
Recommendations for Electric Therapies for Regular Narrow- Complex Tachycardia	1	B-NR
Recommendations for Electric Therapies for Regular Narrow- Complex Tachycardia	1	B-NR

**Recommendations for Pharmacological Therapies for Regular Narrow-Complex Tachycardia**	1	B-R
**Recommendations for Pharmacological Therapies for Regular Narrow-Complex Tachycardia**	1	B-R
**Recommendations for Pharmacological Therapies for Regular Narrow-Complex Tachycardia**	2a	B-R
**Recommendations for Pharmacological Therapies for Regular Narrow-Complex Tachycardia**	2a	C-LD
**Recommendations for Electric Therapies for Atrial Fibrillation/ Flutter**	1	C-LD
**Recommendations for Electric Therapies for Atrial Fibrillation/ Flutter**	1	C-LD

**Recommendations for Electric Therapies for Atrial Fibrillation/ Flutter**	2a	C-LD
**Recommendations for Electric Therapies for Atrial Fibrillation/ Flutter**	2b	C-LD
**Recommendations for Medical Therapies for Atrial Fibrillation/ Flutter**	1	B-NR
**Recommendations for Medical Therapies for Atrial Fibrillation/ Flutter**	2a	B-NR

**Recommendations for Medical Therapies for Atrial Fibrillation/ Flutter**	3: Harm	C-LD
**Recommendations for Medical Therapies for Atrial Fibrillation/ Flutter**	3: Harm	C-EO
Recommendations for Initial Management of arrhythmias other than VF and VT	1	C-EO
Recommendations for Initial Management of arrhythmias other than VF and VT	2a	B-NR

Recommendations for Initial Management of arrhythmias other than VF and VT	2b	C-LD
Recommendations for Initial Management of arrhythmias other than VF and VT	2b	C-EO
**Transvenous Pacing for Bradycardia**	2a	C-LD
**Recommendations for Considerations in the Early Postresuscitation Period**	1	B-NR
**Recommendations for Considerations in the Early Postresuscitation Period**	1	B-NR

**Recommendations for Considerations in the Early Postresuscitation Period**	2a	C-EO
**Blood Pressure Management After ROSC**	2a	B-NR
**Recommendations for Oxygenation and Ventilation After ROSC**	1	B-NR
**Recommendations for Oxygenation and Ventilation After ROSC**	2b	B-R

**Recommendations for Oxygenation and Ventilation After ROSC**	2b	B-R
**Recommendations for Seizure Diagnosis and Management**	1	C-LD
**Recommendations for Seizure Diagnosis and Management**	1	C-LD
**Recommendations for Seizure Diagnosis and Management**	2b	C-LD
**Recommendations for Seizure Diagnosis and Management**	2b	C-LD
**Recommendations for Seizure Diagnosis and Management**	3: No Benefit	B-R
**Recommendations for Other Postresuscitation Care**	2b	B-R

**Recommendations for Other Postresuscitation Care**	2b	B-R
**Recommendations for Other Postresuscitation Care**	2b	B-R
**Recommendations for Other Postresuscitation Care**	2b	B-R
**Recommendations for Indications for TTM**	1	B-R
**Recommendations for Indications for TTM**	1	B-R
**Recommendations for Indications for TTM**	1	B-NR
**Recommendations for Performance of TTM**	1	B-R
**Recommendations for Performance of TTM**	2a	B-NR

**Recommendations for Performance of TTM**	2b	C-LD
**Recommendations for Performance of TTM**	3: No Benefit	A
**Recommendations for PCI After Cardiac Arrest**	1	B-NR
**Recommendations for PCI After Cardiac Arrest**	2a	B-NR
**Recommendations for PCI After Cardiac Arrest**	2a	C-LD
**Recommendations for General Considerations for Neuroprognostication**	1	B-NR

**Recommendations for General Considerations for Neuroprognostication**	1	B-NR
**Recommendations for General Considerations for Neuroprognostication**	1	C-EO
**Recommendations for General Considerations for Neuroprognostication**	2a	B-NR

**Recommendations for Clinical Examination for Neuroprognostication**	2b	B-NR
**Recommendations for Clinical Examination for Neuroprognostication**	2b	B-NR
**Recommendations for Clinical Examination for Neuroprognostication**	2b	B-NR
**Recommendations for Clinical Examination for Neuroprognostication**	2b	B-NR
**Recommendations for Clinical Examination for Neuroprognostication**	2b	B-NR
**Recommendations for Clinical Examination for Neuroprognostication**	3: Harm	B-NR

**Recommendations for Clinical Examination for Neuroprognostication**	3: Harm	B-NR
**Recommendations for Serum Biomarkers for Neuroprognostication**	2b	B-NR
**Recommendations for Serum Biomarkers for Neuroprognostication**	2b	C-LD
**Recommendations for Electrophysiology for Neuroprognostication**	2b	B-NR
**Recommendations for Electrophysiology for Neuroprognostication**	2b	B-NR

**Recommendations for Electrophysiology for Neuroprognostication**	2b	B-NR
**Recommendations for Electrophysiology for Neuroprognostication**	2b	B-NR
**Recommendations for Electrophysiology for Neuroprognostication**	2b	B-NR
**Recommendations for Electrophysiology for Neuroprognostication**	3: No Benefit	B-NR

**Recommendations for Neuroimaging for Neuroprognostication**	2b	B-NR
**Recommendations for Neuroimaging for Neuroprognostication**	2b	B-NR
**Recommendations for Neuroimaging for Neuroprognostication**	2b	B-NR

Recommendations for Accidental Hypothermia	1	C-LD
Recommendations for Accidental Hypothermia	1	C-EO
Recommendations for Accidental Hypothermia	2b	C-LD
Recommendations for Accidental Hypothermia	2b	C-LD

**Recommendations for Anaphylaxis Without Cardiac Arrest**	1	C-LD
**Recommendations for Anaphylaxis Without Cardiac Arrest**	1	C-LD
**Recommendations for Anaphylaxis Without Cardiac Arrest**	1	C-LD
**Recommendations for Anaphylaxis Without Cardiac Arrest**	1	C-LD
**Recommendations for Anaphylaxis Without Cardiac Arrest**	2a	C-LD

**Recommendations for Anaphylaxis Without Cardiac Arrest**	2a	C-LD
**Recommendations for Anaphylaxis Without Cardiac Arrest**	2b	C-LD
**Cardiac Arrest From Anaphylaxis**	1	C-LD
**Recommendations for Management of Cardiac Arrest Due to Asthma**	1	C-LD
**Recommendations for Management of Cardiac Arrest Due to Asthma**	2a	C-LD

**Recommendations for Management of Cardiac Arrest Due to Asthma**	2a	C-LD
**Recommendations for Cardiac Arrest After Cardiac Surgery**	1	B-NR
**Recommendations for Cardiac Arrest After Cardiac Surgery**	1	C-LD
**Recommendations for Cardiac Arrest After Cardiac Surgery**	1	C-EO
**Recommendations for Cardiac Arrest After Cardiac Surgery**	2a	B-NR
**Recommendations for Cardiac Arrest After Cardiac Surgery**	2a	C-LD
**Recommendations for Cardiac Arrest After Cardiac Surgery**	2b	C-LD

**Recommendations for Drowning**	1	C-LD
**Recommendations for Drowning**	1	C-LD
**Recommendations for Drowning**	2b	C-LD
**Recommendations for Drowning**	3: No Benefit	B-NR
**Recommendations for Electrolyte Abnormalities in Cardiac Arrest**	1	C-LD

**Recommendations for Electrolyte Abnormalities in Cardiac Arrest**	1	C-LD
**Recommendations for Electrolyte Abnormalities in Cardiac Arrest**	2b	C-EO
**Recommendations for Electrolyte Abnormalities in Cardiac Arrest**	3: Harm	C-LD
**Recommendations for Acute Management of Opioid Overdose**	1	C-LD
**Recommendations for Acute Management of Opioid Overdose**	1	C-EO

**Recommendations for Acute Management of Opioid Overdose**	1	C-EO
**Recommendations for Acute Management of Opioid Overdose**	2a	B-NR
**Recommendations for Postresuscitation Management of Opioid Overdose**	1	C-LD
**Recommendations for Postresuscitation Management of Opioid Overdose**	2a	C-LD

**Recommendations for Planning and Preparation for Cardiac Arrest in Pregnancy**	1	C-LD
**Recommendations for Planning and Preparation for Cardiac Arrest in Pregnancy**	1	C-LD
**Recommendations for Planning and Preparation for Cardiac Arrest in Pregnancy**	1	C-EO
**Recommendations for Resuscitation of Cardiac Arrest in Pregnancy**	1	C-LD

**Recommendations for Resuscitation of Cardiac Arrest in Pregnancy**	1	C-LD
**Recommendations for Resuscitation of Cardiac Arrest in Pregnancy**	1	C-EO
**Recommendations for Resuscitation of Cardiac Arrest in Pregnancy**	1	C-EO
**Recommendations for Resuscitation of Cardiac Arrest in Pregnancy**	1	C-EO

**Recommendations for Cardiac Arrest and PMCD**	1	C-LD
**Recommendations for Cardiac Arrest and PMCD**	1	C-LD
**Recommendations for Cardiac Arrest and PMCD**	2a	C-EO

**Recommendations for Pulmonary Embolism**	2a	C-LD
**Recommendations for Pulmonary Embolism**	2b	C-LD
**Benzodiazepine Overdose**	3: Harm	B-R
**Recommendations for Calcium Channel Blocker Overdose**	2a	C-LD
**Recommendations for Calcium Channel Blocker Overdose**	2a	C-LD
**Recommendations for Calcium Channel Blocker Overdose**	2b	C-LD

**Recommendations for Calcium Channel Blocker Overdose**	2b	C-LD
**Recommendations for β-Adrenergic Blocker Overdose**	2a	C-LD
**Recommendations for β-Adrenergic Blocker Overdose**	2a	C-LD
**Recommendations for β-Adrenergic Blocker Overdose**	2b	C-LD
**Recommendations for β-Adrenergic Blocker Overdose**	2b	C-LD
**Recommendations for Cocaine Toxicity**	2a	B-NR

**Recommendations for Cocaine Toxicity**	2b	C-LD
**Local Anesthetic Overdose**	2b	C-LD
**Recommendations for Cardiac Arrest Due to Sodium Channel Blockers, Including Tricyclic Antidepressants**	2a	C-LD
**Recommendations for Cardiac Arrest Due to Sodium Channel Blockers, Including Tricyclic Antidepressants**	2b	C-LD
**Recommendations for Carbon Monoxide, Digoxin, and Cyanide Poisoning**	1	B-R

Recommendations for Carbon Monoxide, Digoxin, and Cyanide Poisoning	2b	B-R
Recommendations for Carbon Monoxide, Digoxin, and Cyanide Poisoning	2a	C-LD
Recommendations for Recovery and Survivorship After Cardiac Arrest	1	B-NR
Recommendations for Recovery and Survivorship After Cardiac Arrest	1	C-LD
Recommendations for Recovery and Survivorship After Cardiac Arrest	1	C-LD

**Recommendations for Recovery and Survivorship After Cardiac Arrest**	2b	C-LD

**Explanation**	Topic	Type
1. If a victim is unconscious/unresponsive, with absent or abnormal breathing (ie, only gasping), the lay rescuer should assume the victim is in cardiac arrest.	Initiation of CPR	BLS
2. If a victim is unconscious/unresponsive, with absent or abnormal breathing (ie, only gasping), the healthcare provider should check for a pulse for no more than 10 s and, if no definite pulse is felt, should assume the victim is in cardiac arrest.	Initiation of CPR	BLS
1. All lay rescuers should, at minimum, provide chest compressions for victims of cardiac arrest.	Initiation of CPR	BLS
2. After identifying a cardiac arrest, a lone responder should activate the emergency response system first and immediately begin CPR.	Initiation of CPR	BLS
3. We recommend that laypersons initiate CPR for presumed cardiac arrest, because the risk of harm to the patient is low if the patient is not in cardiac arrest.	Initiation of CPR	BLS
4. For lay rescuers trained in CPR using chest compressions and ventilation (rescue breaths), it is reasonable to provide ventilation (rescue breaths) in addition to chest compressions for the adult in OHCA.	Initiation of CPR	BLS
1. All lay rescuers should, at minimum, provide chest compressions for victims of cardiac arrest.	Initiation of CPR	BLS
2. After identifying a cardiac arrest, a lone responder should activate the emergency response system first and immediately begin CPR.	Initiation of CPR	BLS
3. We recommend that laypersons initiate CPR for presumed cardiac arrest, because the risk of harm to the patient is low if the patient is not in cardiac arrest.	Initiation of CPR	BLS
4. For lay rescuers trained in CPR using chest compressions and ventilation (rescue breaths), it is reasonable to provide ventilation (rescue breaths) in addition to chest compressions for the adult in OHCA.	Initiation of CPR	BLS

1. A lone healthcare provider should commence with chest compressions rather than with ventilation.	Initiation of CPR	BLS
2. It is reasonable for healthcare providers to perform chest compressions and ventilation for all adult patients in cardiac arrest from either a cardiac or noncardiac cause.	Initiation of CPR	BLS
1. A healthcare provider should use the head tilt–chin lift maneuver to open the airway of a patient when no cervical spine injury is suspected.	Airway	BLS
2. The trained lay rescuer who feels confident in performing both compressions and ventilation should open the airway using a head tilt–chin lift maneuver when no cervical spine injury is suspected.	Airway	BLS
3. The use of an airway adjunct (eg, oropharyngeal and/or nasopharyngeal airway) may be reasonable in unconscious (unresponsive) patients with no cough or gag reflex to facilitate delivery of ventilation with a bag-mask device.	Airway	BLS
4. In the presence of known or suspected basal skull fracture or severe coagulopathy, an oral airway is preferred compared with a nasopharyngeal airway.	Airway	BLS
5. The routine use of cricoid pressure in adult cardiac arrest is not recommended.	Airway	BLS

1. In cases of suspected cervical spine injury, healthcare providers should open the airway by using a jaw thrust without head extension.	Airway	BLS
2. In the setting of head and neck trauma, a head tilt–chin lift maneuver should<br>be performed if the airway cannot be opened with a jaw thrust and airway adjunct insertion.	Airway	BLS
3. In the setting of head and neck trauma, lay rescuers should not use<br>immobilization devices because their use by untrained rescuers may be harmful.	Airway	BLS
1. When providing chest compressions, the rescuer should place the heel of one hand on the center (middle) of the victim's chest (the lower half of the sternum) and the heel of the other hand on top of the first so that the hands are overlapped.	Positioning	BLS
2. Resuscitation should generally be conducted where the victim is found, as long as high-quality CPR can be<br>administered safely and effectively in that location.	Positioning	BLS

3. It is preferred to perform CPR on a firm surface and with the victim in the supine position, when feasible.	Positioning	BLS
4. When the victim cannot be placed in the supine position, it may be reasonable for rescuers to provide CPR with the victim in the prone position, particularly in hospitalized patients with an advanced airway in place.	Positioning	BLS
1. In adult cardiac arrest, total preshock and postshock pauses in chest compressions should be as short as possible.	Compressions	BLS
2. The healthcare provider should minimize the time taken to check for a pulse (no more than 10 s) during a rhythm check, and if the rescuer does not definitely feel a pulse, chest compressions should be resumed.	Compressions	BLS
3. When 2 or more rescuers are available, it is reasonable to switch chest compressors approximately every 2 min (or after about 5 cycles of compressions and ventilation at a ratio of 30:2) to prevent decreases in the quality of compressions.	Compressions	BLS

4. It is reasonable to immediately resume chest compressions after shock delivery for adults in cardiac arrest in any setting.	Compressions	BLS
5. For adults in cardiac arrest receiving CPR without an advanced airway, it is reasonable to pause compressions to deliver 2 breaths, each given over 1 s.	Compressions	BLS
6. In adult cardiac arrest, it may be reasonable to perform CPR with a chest compression fraction of at least 60%.	Compressions	BLS
1. During manual CPR, rescuers should perform chest compressions to a depth of at least 2 inches, or 5 cm, for an average adult while avoiding excessive chest compression depths (greater than 2.4 inches, or 6 cm).	Compressions	BLS
2. In adult victims of cardiac arrest, it is reasonable for rescuers to perform chest compressions at a rate of 100 to 120/min.	Compressions	BLS
3. It can be beneficial for rescuers to avoid leaning on the chest between compressions to allow complete chest wall recoil for adults in cardiac arrest.	Compressions	BLS

4. It may be reasonable to perform chest compressions so that chest compression and recoil/ relaxation times are approximately equal.	Compressions	BLS
1. It may be reasonable to use audiovisual feedback devices during CPR for real- time optimization of CPR performance.	CPR feedback, monitoring, checks	BLS
2. It may be reasonable to use physiological parameters such as arterial blood pressure or end-tidal CO_2 when feasible to monitor and optimize CPR quality.	CPR feedback, monitoring, checks	BLS
1. It is reasonable for a rescuer to use mouth-to-nose ventilation if ventilation through the victim's mouth is impossible or impractical.	Ventilation	BLS
2. For a victim with a tracheal stoma who requires rescue breathing, either mouth-to- stoma or face mask (pediatric preferred)– to–stoma ventilation may be reasonable.	Ventilation	BLS
1. For adults in cardiac arrest receiving ventilation, tidal volumes of approximately 500 to 600 mL, or enough to produce visible chest rise, are reasonable.	Ventilation	BLS

2. In patients without an advanced airway, it is reasonable to deliver breaths either by mouth or by using bag-mask ventilation.	Ventilation	BLS
3. When providing rescue breaths, it may be reasonable to give 1 breath over 1 s, take a "regular" (not deep) breath, and give a second rescue breath over 1 s.	Ventilation	BLS
4. Rescuers should avoid excessive ventilation (too many breaths or too large a volume) during CPR.	Ventilation	BLS
1. If an adult victim with spontaneous circulation (ie, strong and easily palpable pulses) requires support of ventilation,<br>it may be reasonable for the healthcare provider to give rescue breaths at a rate of about 1 breath every 6 s, or about 10 breaths per minute.	Ventilation	BLS
1. Before placement of an advanced airway (supraglottic airway or tracheal tube),<br>it is reasonable for healthcare providers to perform CPR with cycles of 30 compressions and 2 breaths.	Ventilation	ALS

2. It may be reasonable for EMS providers to use a rate of 10 breaths per minute (1 breath every 6 s) to provide asynchronous ventilation during continuous chest compressions before placement of an advanced airway.	Ventilation	ALS
3. If an advanced airway is in place, it may be reasonable for the provider to deliver 1 breath every 6 s (10 breaths/min) while continuous chest compressions are being performed.	Ventilation	ALS
4. It may be reasonable to initially use minimally interrupted chest compressions (ie, delayed ventilation) for witnessed shockable OHCA as part of a bundle of care.	Ventilation	ALS
1. Defibrillators (using biphasic or monophasic waveforms) are recommended to treat tachyarrhythmias requiring a shock.	Defibrillation	BLS
2. Based on their greater success in arrhythmia termination, defibrillators using biphasic waveforms are preferred over monophasic defibrillators for treatment of tachyarrhythmias.	Defibrillation	BLS

3. A single shock strategy is reasonable in preference to stacked shocks for defibrillation in the setting of unmonitored cardiac arrest.	Defibrillation	BLS
4. It is reasonable that selection of fixed versus escalating energy levels for subsequent shocks for presumed shock-refractory arrhythmias be based on the specific manufacturer's instructions for that waveform. If this is not known, defibrillation at the maximal dose may be considered.	Defibrillation	BLS
5. If using a defibrillator capable of escalating energies, higher energy for second and subsequent shocks may be considered for presumed shock-refractory arrhythmias.	Defibrillation	BLS
6. In the absence of conclusive evidence that one biphasic waveform is superior to another in termination of VF, it is reasonable to use the manufacturer's recommended energy dose for the first shock. If this is not known, defibrillation at the maximal dose may be considered.	Defibrillation	BLS

1. It is reasonable to place defibrillation paddles or pads on the exposed chest in an anterolateral or anteroposterior position, and to use a paddle or pad electrode diameter more than 8 cm in adults.	Defibrillation	BLS
1. It may be reasonable to use a defibrillator in manual mode as compared with automatic mode depending on the skill set of the operator.	Defibrillation	BLS
1. CPR is recommended until a defibrillator or AED is applied.	Defibrillation	BLS
2. In unmonitored cardiac arrest, it is reasonable to provide a brief prescribed period of CPR while a defibrillator is being obtained and readied for use before initial rhythm analysis and possible defibrillation.	Defibrillation	BLS
3. Immediate defibrillation is reasonable for provider-witnessed or monitored VF/pVT of short duration when a defibrillator is already applied or immediately available.	Defibrillation	BLS

1. It may be reasonable to immediately resume chest compressions after shock administration rather than pause CPR to perform a postshock rhythm check in cardiac arrest patients.	Defibrillation	BLS
1. It may be reasonable to charge a manual defibrillator during chest compressions either before or after a scheduled rhythm analysis.	Defibrillation	BLS
1. The value of artifact-filtering algorithms for analysis of electrocardiogram (ECG) rhythms during chest compressions has not been established.	Defibrillation	BLS
2. The value of VF waveform analysis to guide the acute management of<br>adults with cardiac arrest has not been established.	Defibrillation	BLS
1. The usefulness of double sequential defibrillation for refractory shockable rhythm has not been established.	Defibrillation	BLS

1. The precordial thump may be considered at the onset of a rescuer-witnessed, monitored, unstable ventricular tachyarrhythmia when a defibrillator is not immediately ready for use and is performed without delaying CPR or shock delivery.	Other	BLS
2. The precordial thump should not be used routinely for established cardiac arrest.	Other	BLS
1. Electric pacing is not recommended for routine use in established cardiac arrest.	Other	BLS
1. Fist (percussion) pacing may be considered as a temporizing measure in exceptional circumstances such as witnessed, monitored in-hospital arrest (eg, cardiac catheterization laboratory) for bradyasystole before a loss of consciousness and if performed without delaying definitive therapy.	Other	BLS

1. "Cough" CPR may be considered as a temporizing measure for the witnessed, monitored onset of a hemodynamically significant tachyarrhythmia or bradyarrhythmia before a loss of consciousness without delaying definitive therapy.	Other	BLS
1. It is reasonable for providers to first attempt establishing intravenous access for drug administration in cardiac arrest.	Other	BLS
2. Intraosseous access may be considered if attempts at intravenous access are unsuccessful or not feasible.	Other	BLS
3. In appropriately trained providers, central venous access may be considered if attempts to establish intravenous and intraosseous access are unsuccessful or not feasible.	Other	BLS
4. Endotracheal drug administration may be considered when other access routes are not available.	Other	ALS
1. We recommend that epinephrine be administered for patients in cardiac arrest.	Vasopressors	ALS

2. Based on the protocols used in clinical trials, it is reasonable to administer epinephrine 1 mg every 3 to 5 min for cardiac arrest.	Vasopressors	ALS
3. With respect to timing, for cardiac arrest with a nonshockable rhythm, it is reasonable to administer epinephrine as soon as feasible.	Vasopressors	ALS
4. With respect to timing, for cardiac arrest with a shockable rhythm, it may be reasonable to administer epinephrine after initial defibrillation attempts have failed.	Vasopressors	ALS
5. Vasopressin alone or vasopressin in combination with epinephrine may be considered in cardiac arrest but offers no advantage as a substitute for epinephrine in cardiac arrest.	Vasopressors	ALS
6. High-dose epinephrine is not recommended for routine use in cardiac arrest.	Vasopressors	ALS
1. Amiodarone or lidocaine may be considered for VF/pVT that is unresponsive to defibrillation.	Amiodarone or lidocaine	ALS
2. For patients with OHCA, use of steroids during CPR is of uncertain benefit.	Steroids	ALS

3. Routine administration of calcium for treatment of cardiac arrest is not recommended.	Other pharmacological agents	ALS
4. Routine use of sodium bicarbonate is not recommended for patients in cardiac arrest.	Other pharmacological agents	ALS
5. The routine use of magnesium for cardiac arrest is not recommended.	Other pharmacological agents	ALS
1. If an experienced sonographer is present and use of ultrasound does not interfere with the standard cardiac arrest treatment protocol, then ultrasound may be considered as an adjunct to standard patient evaluation, although its usefulness has not been well established.	Adjuncts to CPR	ALS
2. When supplemental oxygen is available, it may be reasonable to use the maximal feasible inspired oxygen concentration during CPR.	Adjuncts to CPR	ALS
3. An abrupt increase in end-tidal CO_2 may be used to detect ROSC during compressions or when a rhythm check reveals an organized rhythm.	Adjuncts to CPR	ALS
4. Routine measurement of arterial blood gases during CPR has uncertain value.	Adjuncts to CPR	ALS

5. Arterial pressure monitoring by arterial line may be used to detect ROSC during chest compressions or when a rhythm check reveals an organized rhythm.	Adjuncts to CPR	ALS
1. If termination of resuscitation (TOR) is being considered, BLS EMS providers should use the BLS termination of resuscitation rule where ALS is not available or may be significantly delayed.	Termination of Resuscitation	BLS
2. It is reasonable for prehospital ALS providers to use the adult ALS TOR rule to terminate resuscitation efforts in the field for adult victims of OHCA.	Termination of Resuscitation	ALS
3. In a tiered ALS- and BLS-provider system, the use of the BLS TOR rule can avoid confusion at the scene of a cardiac arrest without compromising diagnostic accuracy.	Termination of Resuscitation	BLS

4. In intubated patients, failure to achieve an end-tidal CO_2 of greater than 10 mm Hg by waveform capnography<br>after 20 min of ALS resuscitation may be considered as a component of a multimodal approach to decide when to<br>end resuscitative efforts, but it should not be used in isolation.	Termination of Resuscitation	ALS
5. We suggest against the use of point- of-care ultrasound for prognostication during CPR.	Termination of Resuscitation	ALS
6. In nonintubated patients, a specific end- tidal CO_2 cutoff value at any time during CPR should not be used as an indication to end resuscitative efforts.	Termination of Resuscitation	ALS
1. Either bag-mask ventilation or an advanced airway strategy may be considered during CPR for adult cardiac arrest in any setting depending on the situation and skill set of the provider.	Airway	ALS
1. Frequent experience or frequent retraining is recommended for providers who perform endotracheal intubation.	Airway	ALS

2. If advanced airway placement will interrupt chest compressions, providers may consider deferring insertion of the airway until the patient fails to respond to initial CPR and defibrillation attempts or obtains ROSC.	Airway	ALS
3. Continuous waveform capnography is recommended in addition to clinical assessment as the most reliable method of confirming and monitoring correct placement of an endotracheal tube.	Airway	ALS
4. EMS systems that perform prehospital intubation should provide a program of ongoing quality improvement to<br>minimize complications and track overall supraglottic airway and endotracheal tube placement success rates.	Airway	ALS
1. If an advanced airway is used, a supraglottic airway can be used for adults with OHCA in settings with low tracheal intubation success rates or minimal training opportunities for endotracheal tube placement.	Airway	ALS

2. If an advanced airway is used, either a supraglottic airway or endotracheal intubation can be used for adults with OHCA in settings with high tracheal intubation success rates or optimal training opportunities for endotracheal tube placement.	Airway	ALS
3. If an advanced airway is used in the in-hospital setting by expert providers trained in these procedures, either a supraglottic airway or an endotracheal tube placement can be used.	Airway	ALS
1. The effectiveness of active compression-decompression CPR is uncertain. Active compression-decompression CPR might be considered for use when providers are adequately trained and monitored.	Compressions	ALS
2. The combination of active compression-decompression CPR and impedance threshold device may be reasonable in settings with available equipment and properly trained personnel.	Compressions	ALS

3. The routine use of the impedance threshold device as an adjunct during conventional CPR is not recommended.	Compressions	ALS
1. The use of mechanical CPR devices may be considered in specific settings where the delivery of high-quality manual compressions may be challenging or dangerous for the provider, as long as rescuers strictly limit interruptions in CPR during deployment and removal of the device.	Compressions	ALS
2. The routine use of mechanical CPR devices is not recommended.	Compressions	ALS
1. Interposed abdominal compression CPR may be considered during in-hospital resuscitation when sufficient personnel trained in its use are available.	Compressions	ALS

1. There is insufficient evidence to recommend the routine use of extracorporeal CPR (ECPR) for patients with cardiac arrest. ECPR may be considered for select cardiac arrest patients for whom the suspected cause of the cardiac arrest is potentially reversible during a limited period of mechanical cardiorespiratory support.	ECMO	ALS
1. In hemodynamically stable patients,<br>IV adenosine may be considered for treatment and aiding rhythm diagnosis when the cause of the regular, monomorphic rhythm cannot be determined.	Antiarrytmic agents excluding amidoarone and lidocaine	ALS
2. Administration of IV amiodarone, procainamide, or sotalol may be considered for the treatment of wide- complex tachycardia.	Antiarrytmic agents excluding amidoarone and lidocaine, for WCT	ALS
3. Verapamil should not be administered for any wide-complex tachycardia unless known to be of supraventricular origin<br>and not being conducted by an accessory pathway.	Antiarrytmic agents excluding amidoarone and lidocaine, for WCT	ALS

4. Adenosine should not be administered for hemodynamically unstable, irregularly irregular, or polymorphic wide-complex tachycardias.	Antiarrytmic agents excluding amidoarone and lidocaine, for WCT	ALS
1. If pharmacological therapy is unsuccessful for the treatment of a hemodynamically stable wide-complex tachycardia, cardioversion or seeking urgent expert consultation is reasonable.	Defibrillation	ALS
1. Immediate defibrillation is recommended for sustained, hemodynamically unstable polymorphic VT.	Defibrillation	ALS
1. Magnesium may be considered for treatment of polymorphic VT associated with a long QT interval (torsades de pointes).	Antiarrytmic agents excluding amidoarone and lidocaine, for VT/ VF	ALS
1. IV lidocaine, amiodarone, and measures to treat myocardial ischemia may be considered to treat polymorphic VT in the absence of a prolonged QT interval.	Amiodarone or lidocaine	ALS
2. We do not recommend routine use of magnesium for the treatment of polymorphic VT with a normal QT interval.	Antiarrytmic agents excluding amidoarone and lidocaine, for VT/ VF	ALS
1. Synchronized cardioversion is recommended for acute treatment in patients with hemodynamically unstable SVT.	Management of arrhythmias other than VF and VT	ALS
2. Synchronized cardioversion is recommended for acute treatment in patients with hemodynamically stable SVT when vagal maneuvers and pharmacological therapy is ineffective or contraindicated.	Management of arrhythmias other than VF and VT	ALS

1. Vagal maneuvers are recommended for acute treatment in patients with SVT at a regular rate.	Management of arrhythmias other than VF and VT	ALS
2. Adenosine is recommended for acute treatment in patients with SVT at a regular rate.	Management of arrhythmias other than VF and VT	ALS
3. IV diltiazem or verapamil can be effective for acute treatment in patients with hemodynamically stable SVT at a regular rate.	Management of arrhythmias other than VF and VT	ALS
4. IV β-adrenergic blockers are reasonable for acute treatment in patients with hemodynamically stable SVT at a regular rate.	Management of arrhythmias other than VF and VT	ALS
1. Hemodynamically unstable patients with atrial fibrillation or atrial flutter with rapid ventricular response should receive electric cardioversion.	Management of arrhythmias other than VF and VT	ALS
2. Urgent direct-current cardioversion of new-onset atrial fibrillation in the setting of acute coronary syndrome is recommended for patients with hemodynamic compromise, ongoing ischemia, or inadequate rate control.	Management of arrhythmias other than VF and VT	ALS

3. For synchronized cardioversion of atrial fibrillation using biphasic energy, an initial energy of 120 to 200 J is reasonable, depending on the specific biphasic defibrillator being used.	Management of arrhythmias other than VF and VT	ALS
4. For synchronized cardioversion of atrial flutter using biphasic energy, an initial energy of 50 to 100 J may be reasonable, depending on the specific biphasic defibrillator being used.	Management of arrhythmias other than VF and VT	ALS
1. IV administration of a β-adrenergic blocker or nondihydropyridine calcium channel antagonist is recommended to slow the ventricular heart rate in the acute setting in patients with atrial fibrillation or atrial flutter with rapid ventricular response without preexcitation.	Management of arrhythmias other than VF and VT	ALS
2. IV amiodarone can be useful for rate control in critically ill patients with atrial fibrillation with rapid ventricular response without preexcitation.	Management of arrhythmias other than VF and VT	ALS

3. In patients with atrial fibrillation and atrial flutter in the setting of preexcitation, digoxin, nondihydropyridine calcium channel antagonists, β-adrenergic blockers, and IV amiodarone should not be administered because they may increase the ventricular response and result in VF.	Management of arrhythmias other than VF and VT	ALS
4. Nondihydropyridine calcium channel antagonists and IV β-adrenergic blockers should not be used in patients with left ventricular systolic dysfunction and decompensated heart failure because these may lead to further hemodynamic compromise.	Management of arrhythmias other than VF and VT	ALS
1. In patients presenting with acute symptomatic bradycardia, evaluation and treatment of reversible causes is recommended.	Management of arrhythmias other than VF and VT	ALS
2. In patients with acute bradycardia associated with hemodynamic compromise, administration of atropine is reasonable to increase heart rate.	Management of arrhythmias other than VF and VT	ALS

3. If bradycardia is unresponsive to atropine, IV adrenergic agonists with rate- accelerating effects (eg, epinephrine) or transcutaneous pacing may be effective while the patient is prepared for emergent transvenous temporary pacing if required.	Management of arrhythmias other than VF and VT	ALS
4. Immediate pacing might be considered in unstable patients with high-degree AV block when IV/IO access is not available.	Management of arrhythmias other than VF and VT	ALS
1. In patients with persistent hemodynamically unstable bradycardia refractory to medical therapy, temporary transvenous pacing is reasonable to increase heart rate and improve symptoms.	Management of arrhythmias other than VF and VT	ALS
1. A comprehensive, structured, multidisciplinary system of care should be implemented in a consistent manner for the treatment of post–cardiac arrest patients.	Post ROSC diagnostics	ALS
2. A 12-lead ECG should be obtained as soon as feasible after ROSC to determine whether acute ST-segment elevation is present.	Post ROSC diagnostics	ALS

3. To avoid hypoxia in adults with ROSC in the immediate postarrest period, it is reasonable to use the highest available oxygen concentration until the arterial oxyhemoglobin saturation or the partial pressure of arterial oxygen can be measured reliably.	Post ROSC ventilation	ALS
1. It is preferable to avoid hypotension by maintaining a systolic blood pressure of at least 90 mm Hg and a mean arterial pressure of at least 65 mm Hg in the postresuscitation period.	Post ROSC hemodynamic management	ALS
1. We recommend avoiding hypoxemia in all patients who remain comatose after ROSC.	Post ROSC ventilation	ALS
2. Once reliable measurement of peripheral blood oxygen saturation is available, avoiding hyperoxemia by titrating the fraction of inspired oxygen to target an oxygen saturation of 92% to 98% may be reasonable in patients who remain comatose after ROSC.	Post ROSC ventilation	ALS

3. Maintaining the arterial partial pressure of carbon dioxide ($Paco_2$) within a normal physiological range (generally 35–45 mm Hg) may be reasonable in patients who remain comatose after ROSC.	Post ROSC ventilation	ALS
1. We recommend treatment of clinically apparent seizures in adult post–cardiac arrest survivors.	Post ROSC seizure management	ALS
2. We recommend promptly performing and interpreting an electroencephalogram (EEG) for the diagnosis of seizures in all comatose patients after ROSC.	Post ROSC seizure management	ALS
3. The treatment of nonconvulsive seizures (diagnosed by EEG only) may be considered.	Post ROSC seizure management	ALS
4. The same anticonvulsant regimens used for the treatment of seizures caused by other etiologies may be considered for seizures detected after cardiac arrest.	Post ROSC seizure management	ALS
5. Seizure prophylaxis in adult post–cardiac arrest survivors is not recommended.	Post ROSC seizure management	ALS
1. The benefit of any specific target range of glucose management is uncertain in adults with ROSC after cardiac arrest.	Post ROSC glycemic control	ALS

2. The routine use of prophylactic antibiotics in postarrest patients is of uncertain benefit.	Post ROSC antibiotics	ALS
3. The effectiveness of agents to mitigate neurological injury in patients who remain comatose after ROSC is uncertain.	Post ROSC neuroprotective agents	ALS
4. The routine use of steroids for patients with shock after ROSC is of uncertain value.	Post ROSC steroids	ALS
1. We recommend TTM for adults who do not follow commands after ROSC from OHCA with any initial rhythm.	Post ROSC TTM	ALS
2. We recommend TTM for adults who do not follow commands after ROSC from IHCA with initial nonshockable rhythm.	Post ROSC TTM	ALS
3. We recommend TTM for adults who do not follow commands after ROSC from IHCA with initial shockable rhythm.	Post ROSC TTM	ALS
1. We recommend selecting and maintaining a constant temperature between 32°C and 36°C during TTM.	Post ROSC TTM	ALS
2. It is reasonable that TTM be maintained for at least 24 h after achieving target temperature.	Post ROSC TTM	ALS

3. It may be reasonable to actively prevent fever in comatose patients after TTM.	Post ROSC TTM	ALS
4. We do not recommend the routine use of rapid infusion of cold IV fluids for prehospital cooling of patients after ROSC.	Post ROSC TTM	ALS
1. Coronary angiography should be performed emergently for all cardiac arrest patients with suspected cardiac cause of arrest and ST-segment elevation on ECG.	Coronary angiography	ALS
2. Emergent coronary angiography is reasonable for select (eg, electrically or hemodynamically unstable) adult patients who are comatose after OHCA of suspected cardiac origin but without ST-segment elevation on ECG.	Coronary angiography	ALS
3. Independent of a patient's mental status, coronary angiography is reasonable in all post–cardiac arrest patients for whom coronary angiography is otherwise indicated.	Coronary angiography	ALS
1. In patients who remain comatose after cardiac arrest, we recommend that neuroprognostication involve a multimodal approach and not be based on any single finding.	Post ROSC Neuroprognostication	ALS

2. In patients who remain comatose after cardiac arrest, we recommend that neuroprognostication be delayed until adequate time has passed to ensure avoidance of confounding by medication effect or a transiently poor examination in the early postinjury period.	Post ROSC Neuroprognostication	ALS
3. We recommend that teams caring for comatose cardiac arrest survivors have regular and transparent multidisciplinary discussions with surrogates about the anticipated time course for and uncertainties around neuroprognostication.	Post ROSC Neuroprognostication	ALS
4. In patients who remain comatose after cardiac arrest, it is reasonable to perform multimodal neuroprognostication at a minimum of 72 h after normothermia, though individual prognostic tests may be obtained earlier than this.	Post ROSC Neuroprognostication	ALS

1. When performed with other prognostic tests, it may be reasonable to consider bilaterally absent pupillary light reflex at 72 h or more after cardiac arrest<br>to support the prognosis of poor neurological outcome in patients who remain comatose.	Post ROSC Neuroprognostication	ALS
2. When performed with other prognostic tests, it may be reasonable to consider quantitative pupillometry at 72 h or more after cardiac arrest to support the prognosis of poor neurological outcome in patients who remain comatose.	Post ROSC Neuroprognostication	ALS
3. When performed with other prognostic tests, it may be reasonable to consider bilaterally absent corneal reflexes at 72 h or more after cardiac arrest to support the prognosis of poor neurological outcome in patients who remain comatose.	Post ROSC Neuroprognostication	ALS
4. When performed with other prognostic tests, it may be reasonable to consider status myoclonus that occurs within<br>72 h after cardiac arrest to support the prognosis of poor neurological outcome.	Post ROSC Neuroprognostication	ALS
5. We suggest recording EEG in the presence of myoclonus to determine if there is an associated cerebral correlate.	Post ROSC Neuroprognostication	ALS
6. The presence of undifferentiated myoclonic movements after cardiac arrest should not be used to support a poor neurological prognosis.	Post ROSC Neuroprognostication	ALS

7. We recommend that the findings of a best motor response in the upper extremities being either absent or extensor movements not be used alone for predicting a poor neurological outcome in patients who remain comatose after cardiac arrest.	Post ROSC Neuroprognostication	ALS
1. When performed in combination with other prognostic tests, it may be reasonable to consider high serum values of neuron-specific enolase (NSE) within 72 h after cardiac arrest to support the prognosis of poor neurological outcome in patients who remain comatose.	Post ROSC Neuroprognostication	ALS
2. The usefulness of S100 calcium-binding protein (S100B), Tau, neurofilament light chain, and glial fibrillary acidic protein in neuroprognostication is uncertain.	Post ROSC Neuroprognostication	ALS
1. When evaluated with other prognostic tests, the prognostic value of seizures in patients who remain comatose after cardiac arrest is uncertain.	Post ROSC Neuroprognostication	ALS
2. When performed with other prognostic tests, it may be reasonable to consider persistent status epilepticus 72 h or more after cardiac arrest to support the prognosis of poor neurological outcome.	Post ROSC Neuroprognostication	ALS

3. When performed with other prognostic tests, it may be reasonable to consider burst suppression on EEG in the absence of sedating medications at 72 h or more after arrest to support the prognosis of poor neurological outcome.	Post ROSC Neuroprognostication	ALS
4. When performed with other prognostic tests, it may be reasonable to consider bilaterally absent N20 somatosensory evoked potential (SSEP) waves more than 24 h after cardiac arrest to support the prognosis of poor neurological outcome.	Post ROSC Neuroprognostication	ALS
5. When evaluated with other prognostic tests after arrest, the usefulness of rhythmic periodic discharges to support the prognosis of poor neurological outcome is uncertain.	Post ROSC Neuroprognostication	ALS
6. We recommend that the absence of EEG reactivity within 72 h after arrest not be used alone to support a poor neurological prognosis.	Post ROSC Neuroprognostication	ALS

1. When performed with other prognostic tests, it may be reasonable to consider reduced gray-white ratio (GWR) on brain computed tomography (CT) after cardiac arrest to support the prognosis of poor neurological outcome in patients who remain comatose.	Post ROSC Neuroprognostication	ALS
2. When performed with other prognostic tests, it may be reasonable to consider extensive areas of restricted diffusion on brain MRI (MRI) at 2 to 7 days after cardiac arrest to support the prognosis of poor neurological outcome in patients who remain comatose.	Post ROSC Neuroprognostication	ALS
3. When performed with other prognostic tests, it may be reasonable to consider extensive areas of reduced apparent diffusion coefficient (ADC) on brain MRI at 2 to 7 days after cardiac arrest to support the prognosis of poor neurological outcome in patients who remain comatose.	Post ROSC Neuroprognostication	ALS

1. Full resuscitative measures, including extracorporeal rewarming when available, are recommended for all victims of accidental hypothermia without characteristics that deem them unlikely to survive and without any obviously lethal traumatic injury.	Accidental hypothermia	ALS
2. Victims of accidental hypothermia should not be considered dead before rewarming has been provided unless there are signs of obvious death.	Accidental hypothermia	ALS
3. It may be reasonable to perform defibrillation attempts according to the standard BLS algorithm concurrent with rewarming strategies.	Accidental hypothermia	ALS
4. It may be reasonable to consider administration of epinephrine during cardiac arrest according to the standard ACLS algorithm concurrent with rewarming strategies.	Accidental hypothermia	ALS

1. Epinephrine should be administered early by intramuscular injection (or autoinjector) to all patients with signs of a systemic allergic reaction, especially hypotension, airway swelling, or difficulty breathing.	Anaphylaxis or Asthma	ALS
2. The recommended dose of epinephrine in anaphylaxis is 0.2 to 0.5 mg (1:1000) intramuscularly, to be repeated every 5 to 15 min as needed.	Anaphylaxis or Asthma	ALS
3. In patients with anaphylactic shock, close hemodynamic monitoring is recommended.	Anaphylaxis or Asthma	ALS
4. Given the potential for the rapid development of oropharyngeal or laryngeal edema, immediate referral to a health professional with expertise in advanced airway placement, including surgical airway management, is recommended.	Anaphylaxis or Asthma	ALS
5. When an IV line is in place, it is reasonable to consider the IV route for epinephrine in anaphylactic shock, at a dose of 0.05 to 0.1 mg (0.1 mg/mL, aka 1:10 000).	Anaphylaxis or Asthma	ALS

6. IV infusion of epinephrine is a reasonable alternative to IV boluses for treatment of anaphylaxis in patients not in cardiac arrest.	Anaphylaxis or Asthma	ALS
7. IV infusion of epinephrine may be considered for postarrest shock in patients with anaphylaxis.	Anaphylaxis or Asthma	ALS
1. In cardiac arrest secondary to anaphylaxis, standard resuscitative measures and immediate administration of epinephrine should take priority.	Anaphylaxis or Asthma	ALS
1. For asthmatic patients with cardiac arrest, sudden elevation in peak inspiratory pressures or difficulty ventilating should prompt evaluation for tension pneumothorax.	Anaphylaxis or Asthma	ALS
2. Due to the potential effects of intrinsic positive end-expiratory pressure (auto- PEEP) and risk of barotrauma in an asthmatic patient with cardiac arrest, a ventilation strategy of low respiratory rate and tidal volume is reasonable.	Anaphylaxis or Asthma	ALS

3. If increased auto-PEEP or sudden decrease in blood pressure is noted in asthmatics receiving assisted ventilation in a periarrest state, a brief disconnection from the bag mask or ventilator with compression of the chest wall to relieve air-trapping can be effective.	Anaphylaxis or Asthma	ALS
1. External chest compressions should be performed if emergency resternotomy is not immediately available.	Cardiac arrest in cardiac surgery	ALS
2. In a trained provider-witnessed arrest of a post–cardiac surgery patient,<br>immediate defibrillation for VF/VT should be performed. CPR should be initiated if defibrillation is not successful within 1 min.	Cardiac arrest in cardiac surgery	ALS
3. In a trained provider-witnessed arrest of a post–cardiac surgery patient where pacer wires are already in place, we recommend immediate pacing in an asystolic or bradycardic arrest. CPR should be initiated if pacing is not successful within 1 min.	Cardiac arrest in cardiac surgery	ALS
4. For patients with cardiac arrest after cardiac surgery, it is reasonable to perform resternotomy early in an appropriately staffed and equipped ICU.	Cardiac arrest in cardiac surgery	ALS
5. Open-chest CPR can be useful if cardiac arrest develops during surgery when the chest or abdomen is already open, or<br>in the early postoperative period after cardiothoracic surgery.	Cardiac arrest in cardiac surgery	ALS
6. In post–cardiac surgery patients who are refractory to standard resuscitation procedures, mechanical circulatory support may be effective in improving outcome.	Cardiac arrest in cardiac surgery	ALS

1. Rescuers should provide CPR, including rescue breathing, as soon as an unresponsive submersion victim is removed from the water.	Drowning	ALS
2. All victims of drowning who require any form of resuscitation (including rescue breathing alone) should be transported to the hospital for evaluation and monitoring, even if they appear to be alert and demonstrate effective cardiorespiratory function at the scene.	Drowning	ALS
3. Mouth-to-mouth ventilation in the water may be helpful when administered by a trained rescuer if it does not compromise safety.	Drowning	ALS
4. Routine stabilization of the cervical spine in the absence of circumstances that suggest a spinal injury is not recommended.	Drowning	ALS
1. For cardiac arrest with known or suspected hyperkalemia, in addition to standard ACLS care, IV calcium should be administered.	Electrolyte Abnormalities in Cardiac Arrest	ALS

2. For cardiotoxicity and cardiac arrest from severe hypomagnesemia, in addition to standard ACLS care, IV magnesium is recommended.	Electrolyte Abnormalities in Cardiac Arrest	ALS
3. For cardiac arrest with known or suspected hypermagnesemia, in addition to standard ACLS care, it may be reasonable to administer empirical IV calcium.	Electrolyte Abnormalities in Cardiac Arrest	ALS
4. IV bolus administration of potassium for cardiac arrest in suspected hypokalemia is not recommended.	Electrolyte Abnormalities in Cardiac Arrest	ALS
1. For patients in respiratory arrest, rescue breathing or bag-mask ventilation should be maintained until spontaneous breathing returns, and standard BLS and/ or ACLS measures should continue if return of spontaneous breathing does not occur.	Cardiac arrest due to overdose, toxicity	ALS
2. For patients known or suspected to be in cardiac arrest, in the absence of a proven benefit from the use of naloxone, standard resuscitative measures should take priority over naloxone administration, with a focus on high-quality CPR (compressions plus ventilation).	Cardiac arrest due to overdose, toxicity	ALS

3. Lay and trained responders should not delay activating emergency response systems while awaiting the patient’s response to naloxone or other interventions.	Cardiac arrest due to overdose, toxicity	ALS
4. For a patient with suspected opioid overdose who has a definite pulse but no normal breathing or only gasping (ie, a respiratory arrest), in addition to providing standard BLS and/or ACLS care, it is reasonable for responders to administer naloxone.	Cardiac arrest due to overdose, toxicity	ALS
1. After return of spontaneous breathing, patients should be observed in a healthcare setting until the risk of recurrent opioid toxicity is low and the patient’s level of consciousness and vital signs have normalized.	Cardiac arrest due to overdose, toxicity	ALS
2. If recurrent opioid toxicity develops, repeated small doses or an infusion of naloxone can be beneficial.	Cardiac arrest due to overdose, toxicity	ALS

1. Team planning for cardiac arrest in pregnancy should be done in collaboration with the obstetric, neonatal, emergency, anesthesiology, intensive care, and cardiac arrest services.	Pregnancy	ALS
2. Because immediate ROSC cannot always be achieved, local resources for a perimortem cesarean delivery should be summoned as soon as cardiac arrest in a woman in the second half of pregnancy is recognized.	Pregnancy	ALS
3. Protocols for management of OHCA in pregnancy should be developed to facilitate timely transport to a center with capacity to immediately perform perimortem cesarean delivery while providing ongoing resuscitation.	Pregnancy	ALS
1. Priorities for the pregnant woman in cardiac arrest should include provision of high-quality CPR and relief of aortocaval compression through left lateral uterine displacement.	Pregnancy	ALS

2. Because pregnant patients are more prone to hypoxia, oxygenation and airway management should be prioritized during resuscitation from cardiac arrest in pregnancy.	Pregnancy	ALS
3. Because of potential interference with maternal resuscitation, fetal monitoring should not be undertaken during cardiac arrest in pregnancy.	Pregnancy	ALS
4. We recommend targeted temperature management for pregnant women who remain comatose after resuscitation from cardiac arrest.	Pregnancy	ALS
5. During targeted temperature management of the pregnant patient, it is recommended that the fetus be continuously monitored for bradycardia as a potential complication, and obstetric and neonatal consultation should be sought.	Pregnancy	ALS

1. During cardiac arrest, if the pregnant woman with a fundus height at or above the umbilicus has not achieved ROSC with usual resuscitation measures plus manual left lateral uterine displacement, it is advisable to prepare to evacuate the uterus while resuscitation continues.	Pregnancy	ALS
2. In situations such as nonsurvivable maternal trauma or prolonged pulselessness, in which maternal resuscitative efforts are considered futile, there is no reason to delay performing perimortem cesarean delivery in appropriate patients.	Pregnancy	ALS
3. To accomplish delivery early, ideally within 5 min after the time of arrest, it is reasonable to immediately prepare for perimortem cesarean delivery while initial BLS and ACLS interventions are being performed.	Pregnancy	ALS

1. In patients with confirmed pulmonary embolism as the precipitant of cardiac arrest, thrombolysis, surgical embolectomy, and mechanical embolectomy are reasonable emergency treatment options.	Pulmonary embolism	ALS
2. Thrombolysis may be considered when cardiac arrest is suspected to be caused by pulmonary embolism.	Pulmonary embolism	ALS
1. The administration of flumazenil to patients with undifferentiated coma confers risk and is not recommended.	Cardiac arrest due to overdose, toxicity	ALS
1. In patients with calcium channel blocker overdose who are in refractory shock, administration of calcium is reasonable.	Cardiac arrest due to overdose, toxicity	ALS
2. In patients with calcium channel blocker overdose who are in refractory shock, administration of high-dose insulin with glucose is reasonable.	Cardiac arrest due to overdose, toxicity	ALS
3. In patients with calcium channel blocker overdose who are in refractory shock, administration of IV glucagon may be considered.	Cardiac arrest due to overdose, toxicity	ALS

4. In patients with calcium channel blocker overdose who are in shock refractory to pharmacological therapy, ECMO might be considered.	Cardiac arrest due to overdose, toxicity	ALS
1. In patients with β-adrenergic blocker overdose who are in refractory shock, administration of high-dose insulin with glucose is reasonable.	Cardiac arrest due to overdose, toxicity	ALS
2. In patients with β-adrenergic blocker overdose who are in refractory shock, administration of IV glucagon is reasonable.	Cardiac arrest due to overdose, toxicity	ALS
3. In patients with β-adrenergic blocker overdose who are in refractory shock, administration of calcium may be considered.	Cardiac arrest due to overdose, toxicity	ALS
4. In patients with β-adrenergic blocker overdose who are in shock refractory to pharmacological therapy, ECMO might be considered.	Cardiac arrest due to overdose, toxicity	ALS
1. For patients with cocaine-induced hypertension, tachycardia, agitation, or chest discomfort, benzodiazepines,<br>alpha blockers, calcium channel blockers, nitroglycerin, and/or morphine can be beneficial.	Cardiac arrest due to overdose, toxicity	ALS

2. Although contradictory evidence exists, it may be reasonable to avoid the use of pure β-adrenergic blocker medications in the setting of cocaine toxicity.	Cardiac arrest due to overdose, toxicity	ALS
1. It may be reasonable to administer IV lipid emulsion, concomitant with standard resuscitative care, to patients with local anesthetic systemic toxicity (LAST), and particularly to patients who have premonitory neurotoxicity or cardiac arrest due to bupivacaine toxicity.	Cardiac arrest due to overdose, toxicity	ALS
1. Administration of sodium bicarbonate for cardiac arrest or life-threatening cardiac conduction delays (ie, QRS prolongation more than 120 ms) due to sodium channel blocker/tricyclic antidepressant (TCA) overdose can be beneficial.	Cardiac arrest due to overdose, toxicity	ALS
2. The use of ECMO for cardiac arrest or refractory shock due to sodium channel blocker/TCA toxicity may be considered.	Cardiac arrest due to overdose, toxicity	ALS
1. Antidigoxin Fab antibodies should be administered to patients with severe cardiac glycoside toxicity.	Cardiac arrest due to overdose, toxicity	ALS

2. Hyperbaric oxygen therapy may be helpful in the treatment of acute carbon monoxide poisoning in patients with severe toxicity.	Cardiac arrest due to overdose, toxicity	ALS
3. Hydroxocobalamin and 100% oxygen, with or without sodium thiosulfate, can be beneficial for cyanide poisoning.	Cardiac arrest due to overdose, toxicity	ALS
1. We recommend structured assessment for anxiety, depression, posttraumatic stress, and fatigue for cardiac arrest survivors and their caregivers.	Recovery and survivorship	Recovery
2. We recommend that cardiac arrest survivors have multimodal rehabilitation assessment and treatment for physical, neurological, cardiopulmonary, and cognitive impairments before discharge from the hospital.	Recovery and survivorship	Recovery
3. We recommend that cardiac arrest survivors and their caregivers receive comprehensive, multidisciplinary discharge planning, to include medical and rehabilitative treatment recommendations and return to activity/ work expectations.	Recovery and survivorship	Recovery

4. Debriefings and referral for follow-up for emotional support for lay rescuers, EMS providers, and hospital-based healthcare workers after a cardiac arrest event may be beneficial.	Recovery and survivorship	Recovery

rad	type	topic
1	BLS	Other
2	BLS	Positioning
3	BLS	Drowning
4	BLS	Drowning
5	BLS	Drowning
6	BLS	Drowning
7	BLS	Drowning
8	BLS	Defibrillation
9	BLS	Drowning
10	BLS	Drowning
11	BLS	Drowning
12	BLS	Defibrillation
13	BLS	Initiation of CPR
14	BLS	Ventilation
15	BLS	Compressions
16	BLS	Compressions
17	BLS	Compressions
18	BLS	Defibrillation
19	BLS	CPR feedback, monitoring, checks
20	BLS	Compressions
21	BLS	Compressions
22	BLS	Compressions
23	BLS	Compressions
24	BLS	Adjuncts to CPR
25	BLS	Adjuncts to CPR
26	BLS	Compressions
27	BLS	Compressions
28	BLS	CPR feedback, monitoring, checks
29	BLS	CPR feedback, monitoring, checks
30	BLS	Ventilation
31	BLS	Defibrillation
32	BLS	Compressions
33	BLS	Ventilation
34	BLS	Ventilation
35	BLS	Ventilation
36	BLS	Ventilation
37	BLS	Ventilation
38	BLS	Ventilation
39	BLS	Ventilation
40	BLS	Ventilation
41	BLS	Ventilation

42	BLS	Compressions
43	BLS	Compressions
44	BLS	Compressions
45	BLS	Compressions
46	BLS	Compressions
47	BLS	Compressions
48	BLS	Compressions
49	BLS	Compressions
50	BLS	Compressions
51	BLS	Compressions
52	BLS	Ventilation
53	BLS	Compressions
54	BLS	Compressions
55	BLS	Compressions
56	BLS	Adjuncts to CPR
57	BLS	Adjuncts to CPR
58	BLS	Cardiac arrest due to overdose, toxicity
59	BLS	Termination of Resuscitation
60	BLS	Adjuncts to CPR
61	ALS	Coronary angiography
62	ALS	Coronary angiography
63	ALS	Defibrillation
64	ALS	Defibrillation
65	ALS	Defibrillation
66	ALS	Defibrillation
67	ALS	Other
68	ALS	Other
69	ALS	Other
70	ALS	Other
71	ALS	Ventilation
72	ALS	Steroids
73	ALS	Adjuncts to CPR
74	ALS	Ventilation
75	ALS	Anaphylaxis or Asthma
76	ALS	ECMO
77	ALS	Steroids
78	ALS	Ventilation
79	ALS	Ventilation
80	ALS	Neuroprognostication
81	ALS	Neuroprognostication
82	ALS	Neuroprognostication
83	ALS	Neuroprognostication

84	ALS	Neuroprognostication
85	ALS	Neuroprognostication
86	ALS	Neuroprognostication
87	ALS	Neuroprognostication
88	ALS	Neuroprognostication
89	ALS	Neuroprognostication
90	ALS	Neuroprognostication
91	ALS	Neuroprognostication
92	ALS	Neuroprognostication
93	ALS	Neuroprognostication
94	ALS	Neuroprognostication
95	ALS	Neuroprognostication

heading
Video-based dispatch system (new: SysRev)
Head-up cardiopulmonary resuscitation (CPR) (new: SysRev)
Bystander CPR in drowning (BLS 856: ScopRev)
In-water resuscitation in drowning (BLS 856: ScopRev)
Resuscitation on a boat after drowning (BLS 856: ScopRev)
Airway management in drowning (BLS 856: ScopRev)
Prehospital oxygen in drowning (BLS 856: ScopRev)
Automated external defibrillator (AED) use in drowning (BLS 856: ScopRev)
Mechanical ventilation in drowning (BLS 856: ScopRev)
Extracorporeal membrane oxygenator (ECMO) in drowning (BLS 856:
Criteria for discharge in drowning (BLS 856: ScopRev)
Paddle size and placement for defibrillation (new: EvUp)
CPR before call for help (BLS 1527: EvUp)
Barrier devices (BLS 342: EvUp)
Chest compression rate (BLS 343: EvUp)
Rhythm check timing (BLS 345: EvUp)
Timing of CPR cycles (2 minutes versus other) (BLS 346: EvUp)
Public-access AED programs (BLS 347: EvUp)
Check for circulation during basic life support (BLS) (BLS 348: EvUp)
Rescuer fatigue in chest compression–only CPR (BLS 349: EvUp)
Harm from CPR to victims not in arrest (BLS 353: EvUp)
Harm to rescuers from CPR (BLS 354: EvUp)
Hand position during compressions (BLS 357: EvUp)
Dispatcher instructions (BLS 359: EvUp)
Dispatcher instructions (BLS 359: EvUp)
Emergency medical services (EMS) chest compression–only CPR versus
Emergency medical services (EMS) chest compression–only CPR versus
Feedback for CPR quality (BLS 361: EvUp)
Feedback for CPR quality (BLS 361: EvUp)
Compression-to-ventilation ratio (BLS 362: EvUp)
CPR before defibrillation (BLS 363: EvUp)
Chest compression depth (BLS 366: EvUp)
Chest wall recoil (BLS 367: EvUp)
Foreign body airway obstruction (BLS 368: EvUp)
Foreign body airway obstruction (BLS 368: EvUp)
Foreign body airway obstruction (BLS 368: EvUp)
Foreign body airway obstruction (BLS 368: EvUp)
Foreign body airway obstruction (BLS 368: EvUp)
Foreign body airway obstruction (BLS 368: EvUp)
Foreign body airway obstruction (BLS 368: EvUp)
Foreign body airway obstruction (BLS 368: EvUp)

Firm surface for CPR (BLS 370: EvUp)
Firm surface for CPR (BLS 370: EvUp)
Firm surface for CPR (BLS 370: EvUp)
Firm surface for CPR (BLS 370: EvUp)
Analysis of rhythm during chest compression (BLS 373: EvUp)
Alternative compression techniques (cough, precordial thump, fist pacing)
Alternative compression techniques (cough, precordial thump, fist pacing)
Alternative compression techniques (cough, precordial thump, fist pacing)
Alternative compression techniques (cough, precordial thump, fist pacing)
Alternative compression techniques (cough, precordial thump, fist pacing)
Tidal volumes and ventilation rates (BLS 546: EvUp)
Lay rescuer chest compression–only CPR versus standard CPR (BLS 547:
Lay rescuer chest compression–only CPR versus standard CPR (BLS 547:
Starting CPR (compression-airway-breathing compared with airway-
Dispatcher recognition of cardiac arrest (BLS 740: EvUp)
Dispatcher recognition of cardiac arrest (BLS 740: EvUp)
Resuscitation care for suspected opioid-associated emergencies (BLS 811: EvUp)
Drowning (BLS 856: EvUp)
Dispatcher-assisted continuous chest compressions CPR versus
Early coronary angiography (CAG) after return of spontaneous circulation
Early coronary angiography (CAG) after return of spontaneous circulation
CPR and defibrillation in the prone patient (new: SysRev)
CPR and defibrillation in the prone patient (new: SysRev)
CPR and defibrillation in the prone patient (new: SysRev)
CPR and defibrillation in the prone patient (new: SysRev)
Consciousness during CPR (new: ScopRev)
Consciousness during CPR (new: ScopRev)
Consciousness during CPR (new: ScopRev)
Transition from shockable to nonshockable rhythm (ALS 444: EvUp)
Oxygen dose during CPR (ALS 889: EvUp)
Steroids during CPR (ALS 433: EvUp)
Confirmation of tracheal tube position (ALS 469: EvUp)
Automatic ventilators versus manual ventilation during CPR (ALS 490:
Cardiac arrest and asthma (ALS 492: EvUp)
Extracorporeal CPR (ECPR) versus manual or mechanical CPR (ALS 723:
Steroids after ROSC (ALS 446: EvUp)
Oxygen dose after ROSC (ALS 448: EvUp)
Oxygen dose after ROSC (ALS 448: EvUp)
Neuroprognostication after ROSC (ALS 450, 458, 460, 484, 487, 713:
Neuroprognostication after ROSC (ALS 450, 458, 460, 484, 487, 713:
Neuroprognostication after ROSC (ALS 450, 458, 460, 484, 487, 713:
Neuroprognostication after ROSC (ALS 450, 458, 460, 484, 487, 713:

Neuroprognostication after ROSC (ALS 450, 458, 460, 484, 487, 713:
Neuroprognostication after ROSC (ALS 450, 458, 460, 484, 487, 713:
Neuroprognostication after ROSC (ALS 450, 458, 460, 484, 487, 713:
Neuroprognostication after ROSC (ALS 450, 458, 460, 484, 487, 713:
Neuroprognostication after ROSC (ALS 450, 458, 460, 484, 487, 713:
Neuroprognostication after ROSC (ALS 450, 458, 460, 484, 487, 713:
Neuroprognostication after ROSC (ALS 450, 458, 460, 484, 487, 713:
Neuroprognostication after ROSC (ALS 450, 458, 460, 484, 487, 713:
Neuroprognostication after ROSC (ALS 450, 458, 460, 484, 487, 713:
Neuroprognostication after ROSC (ALS 450, 458, 460, 484, 487, 713:
Neuroprognostication after ROSC (ALS 450, 458, 460, 484, 487, 713:
Neuroprognostication after ROSC (ALS 450, 458, 460, 484, 487, 713:

text
We suggest that the usefulness of video-based dispatch systems be assessed in clinical trials or resea
We suggest against the routine use of head-up CPR during CPR (weak recommendation, very low–ce
In the meantime, we highlight our 2020 recommendation and suggest that bystanders who are trained
The 2005 treatment recommendation is unchanged: In-water, expired-air resuscitation may be conside
In the meantime, we highlight our 2020 recommendation and suggest that bystanders who are trained
The lack of evidence in the drowning setting supports the use of standard ALS Task Force recommenc
The lack of evidence for a different approach to prehospital oxygen therapy in the drowning setting su
In the meantime, we highlight our 2020 recommendation suggesting that delivery of a shock with an A
The lack of evidence in the drowning setting supports the use of standard general recommendations f
The evidence identified supports the ILCOR treatment recommendation that states "ECPR may be co
There was no treatment recommendation on criteria for discharge after submersion; a SysRev will be
It is reasonable to place pads on the exposed chest in an anterior-lateral position. An acceptable alter
We recommend that a lone bystander with a mobile phone should dial EMS, activate the speaker or o
Providers should take appropriate safety precautions when feasible and when resources are available
We recommend a manual chest compression rate of 100–120/min (strong recommendation, very low–
We suggest against the checking of cardiac rhythm immediately after defibrillation (weak recommenda
We suggest pausing chest compressions every 2 min to assess the cardiac rhythm (weak recommend
We recommend the implementation of public-access defibrillation programs for patients with OHCAs (
Outside of the ALS environment where invasive monitoring is available, there are insufficient data on t not make a treatment recommendation on the value of a pulse check.
No treatment recommendation
We recommend that laypersons initiate CPR for presumed cardiac arrest without concerns of harm to
Evidence supporting rescuer safety during CPR is limited. The few isolated reports of adverse effects
This treatment recommendation is unchanged from 2015: We suggest performing chest compressions
We recommend that emergency medical dispatch centers have systems in place to enable call handle
We recommend that emergency medical call takers provide CPR instructions (when deemed necessa
We recommend that EMS providers perform CPR with 30 compressions to 2 breaths (30:2 ratio) or co
We suggest that when EMS systems have adopted minimally interrupted cardiac resuscitation, this st
We suggest the use of real-time audiovisual feedback and prompt devices during CPR in clinical pract
We suggest against the use of real-time audiovisual feedback and prompt devices in isolation (ie, not
We suggest a CV ratio of 30:2 compared with any other CV ratio in patients with cardiac arrest (weak
We suggest a short period of CPR until the defibrillator is ready for analysis or defibrillation in unmonit
We recommend a chest compression depth of ≈5 cm (2 in) (strong recommendation, low-quality evide
We suggest that rescuers performing manual CPR avoid leaning on the chest between compressions
We suggest that back slaps are used initially in adults and children with an FBAO and an ineffective c
We suggest that abdominal thrusts are used in adults and children (>1 y of age) with an FBAO and an
We suggest that rescuers consider the manual extraction of visible items in the mouth (weak recomme
We suggest against the use of blind finger sweeps in patients with an FBAO (weak recommendation,
We suggest that appropriately skilled health care providers use Magill forceps to remove an FBAO in
We suggest that chest thrusts be used in unconscious adults and children with an FBAO (weak recom
We suggest that bystanders undertake interventions to support FBAO removal as soon as possible af
We suggest against the routine use of suction-based airway clearance devices (weak recommendatio

We suggest performing manual chest compressions on a firm surface when possible (weak recommen
During IHCA, we suggest that when a bed has a CPR mode that increases mattress stiffness, it shoul
During IHCA, we suggest against moving a patient from a bed to the floor to improve chest compressi
The confidence in effect estimates is so low that the task force was unable to make a recommendatio
We suggest against the routine use of artifact-filtering algorithms for analysis of electrocardiographic r
We recommend against the routine use of cough CPR for cardiac arrest (strong recommendation, ver
We suggest that cough CPR may be considered only as a temporizing measure in exceptional circum
We recommend against fist pacing for cardiac arrest (strong recommendation, very low–certainty evid
We suggest that fist pacing may be considered only as a temporizing measure in the exceptional circu
We recommend against the use of a precordial thump for cardiac arrest (strong recommendation, ver
For mouth-to-mouth ventilation for adult victims using exhaled air or bag-mask ventilation with room a
We continue to recommend that bystanders perform chest compressions for all patients in cardiac arr
We suggest that bystanders who are trained, able, and willing to give rescue breaths and chest comp
We suggest starting CPR with compressions rather than ventilation (weak recommendation, very low–
We recommend that dispatch centers implement a standardized algorithm or standardized criteria to d
We suggest that dispatch centers monitor and track diagnostic capability. We suggest that dispatch ce
We suggest that CPR be started without delay in any unconscious person not breathing normally and respiratory or circulatory arrest (weak recommendation based on expert consensus).
We recommend that submersion duration be used as a prognostic indicator when making decisions o
We recommend that dispatchers provide chest compression–only CPR instructions to callers for adult
When CAG is considered for comatose postarrest patients without ST-segment elevation, we suggest
We suggest early CAG in comatose post–cardiac arrest patients with ST-segment elevation (good pra
For patients with cardiac arrest occurring while in the prone position with an advanced airway already
Invasive blood pressure monitoring and continuous ETCO2 monitoring may be useful to ascertain whe
For patients with cardiac arrest occurring while in the prone position without an advanced airway alre
For patients with cardiac arrest with a shockable rhythm who are in the prone position and cannot be
In settings in which it is feasible, rescuers may consider using sedative or analgesic drugs (or both) in
Neuromuscular-blocking drugs alone should not be given to conscious patients (good practice statem
The optimal drug regimen for sedation and analgesia during CPR is uncertain. Regimens can be base
None
We suggest using the highest possible inspired oxygen concentration during CPR (weak recommenda
For IHCA, the task force was unable to reach a consensus recommendation for or against the use of
We recommend using waveform capnography to confirm and continuously monitor the position of a tr
There is insufficient evidence to support or refute the use of an automatic transport ventilator over ma
There is insufficient evidence to suggest any routine change to cardiac arrest resuscitation treatment
We suggest that ECPR may be considered as a rescue therapy for selected patients with cardiac arre
There is insufficient evidence to support or refute the use of corticosteroids alone or in combination wi
We recommend avoiding hypoxemia in adults with ROSC after cardiac arrest in any setting (strong re
We suggest avoiding hyperoxemia in adults with ROSC after cardiac arrest in any setting (weak recom
We recommend that neuroprognostication always be undertaken with a multimodal approach becaus
Clinical examination: We suggest using PLR at ≥72 h after ROSC for predicting neurological outcome
We suggest using quantitative pupillometry at ≥72 h after ROSC for predicting neurological outcome o
We suggest using bilateral absence of corneal reflex at ≥72 h after ROSC for predicting poor neurolog

We suggest using presence of myoclonus or status myoclonus within 7 d after ROSC, in combination [illegible]
We also suggest recording EEG in the presence of myoclonic jerks to detect any associated epileptifo[illegible]
Electrophysiology: We suggest using a bilaterally absent N20 wave of SSEP in combination with other [illegible]
We suggest against using the absence of EEG background reactivity alone to predict poor outcome in [illegible]
We suggest using the presence of seizure activity on EEG in combination with other indices to predict [illegible]
We suggest using burst suppression on EEG in combination with other indices to predict poor outcom[illegible]
Serum biomarkers: We suggest using NSE within 72 h after ROSC, in combination with other tests, fo[illegible]
There is no consensus on a threshold value. We suggest against using S-100B protein for predicting [illegible]
We suggest against using serum levels of glial fibrillary acidic protein, serum tau protein, or neurofilam[illegible]
Neuroimaging: We suggest using GWR on brain computed tomography for predicting neurological out[illegible]
We suggest using diffusion-weighted brain MRI for predicting neurological outcome of adults who are [illegible]
We suggest using ADC on brain MRI for predicting neurological outcome of adults who are comatose [illegible]

rec	loe
weak	very low–certainty evidence
weak	very low–certainty evidence
weak	very low–certainty evidence
weak	very low–certainty evidence
	very low–certainty evidence
strong	very low–certainty evidence
strong	very low-quality evidence
weak	very low–certainty evidence
weak	low–certainty evidence
strong	low–certainty evidence
strong	very low–certainty evidence
weak	very low–certainty evidence
strong	very low–certainty evidence
strong	very low–certainty evidence
strong	high-certainty evidence
weak	very low–certainty evidence
weak	very low–certainty evidence
weak	very low–certainty evidence
weak	very low-quality evidence
weak	low–certainty evidence
weak	low-quality evidence
weak	very low-quality evidence
weak	very low-quality evidence
weak	very low–certainty evidence
weak	very low–certainty evidence
weak	very low–certainty evidence
weak	very low–certainty evidence
weak	very low–certainty evidence
weak	very low–certainty evidence
weak	very low–certainty evidence

weak	very low–certainty evidence
weak	very low–certainty evidence
weak	very low–certainty evidence
weak	very low-certainty evidence
strong	very low-certainty evidence
weak	very low-certainty evidence
strong	very low-certainty evidence
weak	very low-certainty evidence
strong	very low-certainty evidence
good practice statement	
weak	very low-certainty evidence
weak	very low-certainty evidence
strong	very low-certainty evidence
weak	expert opinion
weak	very low-certainty evidence
strong	low-quality evidence
weak	low-certainty evidence
good practice statement	
good practice statement	
good practice statement	
strong	very low-certainty evidence
good practice statement	
good practice statement	
good practice statement	
good practice statement	
strong	very low-certainty evidence
weak	very low-certainty evidence
strong	low-quality evidence
weak	very low-certainty evidence
strong	very low-certainty evidence
weak	low-certainty evidence
weak	very low-certainty evidence
weak	very low-certainty evidence
weak	low-certainty evidence
weak	very low-certainty evidence

weak	very low-certainty evidence
weak	very low-certainty evidence
weak	very low-certainty evidence
weak	very low-certainty evidence
weak	very low-certainty evidence
weak	very low-certainty evidence
weak	very low-certainty evidence
weak	low-certainty evidence
weak	very low-certainty evidence
weak	very low-certainty evidence
weak	very low-certainty evidence
weak	very low-certainty evidence
weak	

rad	type	topic
1	BLS	Ventilation
2	BLS	Compressions
3	BLS	Compressions
4	BLS	Compressions
5	BLS	Other
6	BLS	Other
7	BLS	Other
8	BLS	Drowning
9	BLS	Drowning
10	BLS	Defibrilllation
11	BLS	Ventilation
12	BLS	Compressions
13	BLS	Compressions
14	BLS	Compressions
15	BLS	Defibrilllation
16	BLS	CPR feedback, monitoring, checks
17	BLS	Compressions
18	BLS	Compressions
19	BLS	Compressions
20	BLS	Compressions
21	BLS	Compressions
22	BLS	Compressions
23	BLS	Compressions
24	BLS	Compressions
25	BLS	Compressions
26	BLS	Compressions
27	BLS	Ventilation
28	BLS	Airway
29	BLS	Compressions
30	BLS	Compressions
31	BLS	Adjuncts to CPR
32	BLS	Compressions
33	BLS	Ventilation
34	BLS	Ventilation
35	BLS	Compressions
36	BLS	Compressions
37	BLS	Adjuncts to CPR
38	BLS	Adjuncts to CPR
39	BLS	Cardiac arrest in special circumsta
40	BLS	BLS recognition and initiation of C
41	BLS	Other
42	BLS	Positioning
43	ALS	TTM
44	ALS	TTM
45	ALS	TTM
46	ALS	TTM

47	ALS	TTM
48	ALS	TTM
49	ALS	TTM
50	ALS	Adjuncts to CPR
51	ALS	Adjuncts to CPR
52	ALS	Adjuncts to CPR
53	ALS	Vasopressors
54	ALS	Vasopressors
55	ALS	Coronary angiography
56	ALS	Coronary angiography
57	ALS	Vasopressors
58	ALS	Pulmonary embolism

heading
Passive ventilation techniques (SysRev)
Minimizing pauses in chest compressions (SysRev)
Minimizing pauses in chest compressions (SysRev)
Minimizing pauses in chest compressions (SysRev)
Cardiopulmonary resuscitation (CPR) during transport (SysRev)
Cardiopulmonary resuscitation (CPR) during transport (SysRev)
Cardiopulmonary resuscitation (CPR) during transport (SysRev)
Compressions-airway-breaths (C-A-B) or airway-breaths-compressions (A-B-C) in drowning (new
Compressions-airway-breaths (C-A-B) or airway-breaths-compressions (A-B-C) in drowning (new
Paddle size and placement for defibrillation (EvUp)
Barrier devices (EvUp)
Chest compression rate (EvUp)
Rhythm check timing (EvUp)
Timing of CPR cycles (2 minutes versus other; EvUp)
Public-access automated external defibrillator (AED) programs (EvUp)
Checking for circulation during basic life support (BLS; EvUp)
Rescuer fatigue in compression-only CPR (EvUp)
Harm from CPR to subjects not in cardiac arrest (EvUp)
Harm to rescuers from CPR (EvUp)
Hand positioning during compressions (EvUp)
Dispatch-assisted compression-only versus conventional CPR (EvUp)
Emergency medical services chest compression–only versus conventional CPR (EvUp)
Compression-to-ventilation ratio (EvUp)
CPR before defibrillation (EvUp)
CPR before defibrillation (EvUp)
Chest compression depth (EvUp)
Chest wall recoil (EvUp)
Foreign body airway obstruction (EvUp)
Firm surface for CPR (EvUp)
In-hospital chest compression–only CPR versus conventional CPR (EvUp)
Analysis of rhythm during chest compressions (EvUp)
Alternative compression techniques (cough, precordial thump, fist pacing; EvUp)
Tidal volumes and ventilation rates (EvUp)
Tidal volumes and ventilation rates (EvUp)
Lay rescuer chest compression–only versus conventional CPR (EvUp)
Starting CPR (C-A-B versus A-C-B; EvUp)
Dispatcher recognition of cardiac arrest (EvUp)
Dispatcher recognition of cardiac arrest (EvUp)
Resuscitation care for suspected opioid-associated emergencies (EvUp)
CPR before call for help (EvUp)
Video-based dispatch (EvUp)
Head-up CPR (EvUp)
Targeted temperature management (TTM) after cardiac arrest (SysRev)
Targeted temperature management (TTM) after cardiac arrest (SysRev)
Targeted temperature management (TTM) after cardiac arrest (SysRev)
Targeted temperature management (TTM) after cardiac arrest (SysRev)

Targeted temperature management (TTM) after cardiac arrest (SysRev)
Targeted temperature management (TTM) after cardiac arrest (SysRev)
Targeted temperature management (TTM) after cardiac arrest (SysRev)
Point-of-care ultrasound (POCUS) as a diagnostic tool during cardiac arrest (SysRev)
Point-of-care ultrasound (POCUS) as a diagnostic tool during cardiac arrest (SysRev)
Point-of-care ultrasound (POCUS) as a diagnostic tool during cardiac arrest (SysRev)
Vasopressin and corticosteroids for cardiac arrest (SysRev)
Vasopressin and corticosteroids for cardiac arrest (SysRev)
Post–cardiac arrest coronary angiography (CAG; SysRev Update)
Post–cardiac arrest coronary angiography (CAG; SysRev Update)
Vasopressors during cardiac arrest (EvUp)
Cardiac arrest from pulmonary embolism (EvUp)

text
We suggest against the routine use of passive ventilation techniques during conventional CPR (weak recommen
We suggest that CPR fraction and perishock pauses in clinical practice be monitored as part of a comprehensive
We suggest that preshock and postshock pauses in chest compressions be as short as possible (weak recommen
We suggest that the CPR fraction during cardiac arrest (CPR time devoted to compressions) should be as high as
We suggest that providers deliver resuscitation at the scene rather than undertake ambulance transport with o
The quality of manual CPR may be reduced during transport. We recommend that whenever transport is indicat
Delivery of manual CPR during transport increases the risk of injury to providers. We recommend that emergen
We recommend a compression-first strategy (C-A-B) for laypeople providing resuscitation for adults and childre
We recommend that health care professionals and those with a duty to respond to drowning (eg, lifeguards) co
No new studies identified
No new studies identified
PICOSTs BLS 343, 366, and 367 have been evaluated together to identify any evidence looking at the interplay b
No new studies identified
No new studies identified
One observational study on a PAD program at Tokyo railroad stations presented significant benefits and cost-eff
No new studies since 2021. Some relevant articles showing the effectiveness of ultrasound to check for circulati
No new clinical or simulation studies were identified that addressed the criteria. Simulation studies on manikins
No new studies identified
One study found low risk of physical injury reported by volunteer citizen responders dispatched to OHCA. One s
No new studies addressing this question were identified, but 2 simulation/training studies highlighting difficulti
No new studies identified
One new study since 2021 was identified. Median inspiratory tidal volume generated by manual chest compress
No new studies identified
No new studies identified
Observational data exploring AMSA and $ETCO_2$ to guide defibrillation might be relevant for ALS.
PICOSTs BLS 343, 366, and 367 have been evaluated together to identify any evidence looking at the interplay a
PICOSTs BLS 343, 366, and 367 have been evaluated together to identify any evidence looking at the interplay a
A single new case series was identified that describes 8 cases of the use of a vacuum cleaner to clear foreign-bo
Three additional manikin RCTs were identified, evaluating CPR quality with a backboard, on a dentist chair, and
No new studies identified
Two new observational studies since last SysRev were identified. Analysis during CPR led to fewer pauses in che
No new studies identified
No new studies identified
Identified studies evaluated tidal volumes during mechanical ventilation and after ROSC.
Only manikin/training studies since 2020
No new studies identified
One RCT was identified in which calls processed with machine learning recognized arrest 93.1% vs 90.5% in control grou
Six observational studies evaluated various interventions or compared different systems with regard to recogni
No new studies identified
No new studies identified
Two additional observational studies were identified. One study reported an association between video dispatc
No new studies identified Observational data exploring AMSA and $ETCO_2$ to guide defibrillation might be relevant for ALS.
We suggest actively preventing fever by targeting a temperature ≤37.5° C for patients who remain comatose aft
Whether subpopulations of cardiac arrest patients may benefit from targeting hypothermia at 32° C to 34° C re
Comatose patients with mild hypothermia after ROSC should not be actively warmed to achieve normothermia
We recommend against the routine use of prehospital cooling with rapid infusion of large volumes of cold intra

We suggest surface or endovascular temperature control techniques when temperature control is used in coma
When a cooling device is used, we suggest using a temperature control device that includes a feedback system l
We suggest active prevention of fever for at least 72 hours in post–cardiac arrest patients who remain comatose
We suggest against routine use of POCUS during CPR to diagnose reversible causes of cardiac arrest (weak recor
We suggest that if POCUS can be performed by experienced personnel without interrupting CPR, it may be cons
Any deployment of diagnostic POCUS during CPR should be carefully considered and weighed against the risks c
We suggest against the use of the combination of vasopressin and corticosteroids in addition to usual care for a
We suggest against the use of the combination of vasopressin and corticosteroids in addition to usual care for a
When CAG is considered for comatose postarrest patients without ST-segment elevation, we suggest that either
We suggest early CAG in comatose post–cardiac arrest patients with ST-segment elevation (good practice stater
Studies support the effect of survival but uncertain effect on functional outcome. Observational studies continu
Small studies that do not change management; there is a need for an EvUp focusing on ECPR for cardiac arrest f

rec	loe
weak	
weak	
weak	
weak	
weak	
strong	
good practice statement	
good practice statement	
good practice statement	
weak	low-certainty
good practice statement	
strong	moderate-ce

weak	low-certainty
good practice statement	
good practice statement	
weak	very low-cert
weak	very low-cert
good practice statement	
weak	low- to mode
weak	very low– to
weak	low-certainty
good practice statement	
