# Supplementary material for: The evidence supporting AHA guidelines on adult cardiopulmonary resuscitation (CPR)
Source: PLoS One. 2024 Dec 17;19(12):e0309241. doi: 10.1371/journal.pone.0309241 (PMC11651587; doi:10.1371/journal.pone.0309241)
Supplement: Attachment — Submitted filename: Response to Reviewers.pdf [file pone.0309241.s003.pdf]

Re: Manuscript ID: PONE-D-24-02668

Title: "The Evidence Supporting AHA Guidelines on Adult Cardiopulmonary Resuscitation (CPR)"

Dear Editorial Team,

Thank you for the opportunity to revise our manuscript in response to the reviewers' comments. We appreciate the thorough review and constructive feedback provided by the reviewers. In this response letter, we address each of the points raised by the academic editor and reviewers.

Reviewer #1:

Reviewer #1 raised concerns regarding the clarity of our methodology and the necessity for a more comprehensive analysis of CPR guidelines. In response, we have revised the methodology section to provide a clearer explanation of the guidelines' collection and categorization process. Additionally, to enhance clarity, we have included a separate document containing all guidelines in their entirety, illustrating that the only processing of the guidelines performed was their categorization into ALS/BLS/Recovery, along with the assignment of each guideline to a specific topic for streamlined data presentation.

Due to the absence of updates to guidelines post-2020, we were unable to incorporate these subsequent revisions into our analysis. Furthermore, we have enhanced the discussion section to offer a more nuanced interpretation of our findings and their implications for future research.

Reviewer #2:

Thoroughness of Analysis: We have addressed the reviewer's request for additional context regarding the relevance of the guidelines and a more detailed explanation of the guideline selection process. In the revised manuscript, we have included a clear description of the topics covered by the guidelines and the rationale behind our categorization process. We have also provided information on how the guideline recommendations were reviewed and assessed for strength and evidence level.

Clarity and Completeness: We have incorporated the reviewer's suggestions for improving the clarity and completeness of our manuscript. Specifically, we have included explanations of the context in which the guidelines are relevant, details on the guideline review process, and information on any modifications to the guidelines over time. Additionally, we have addressed each of the minor corrections pointed out by the reviewer.

Reviewer #3:

Insightfulness of Submission: We appreciate the reviewer's positive feedback on the insightfulness of our submission. We have carefully considered the reviewer's suggestions for minor revisions and have incorporated them into the revised manuscript.

Additional Suggestions: We have addressed the reviewer's additional suggestions, including clarifications in the abstract, corrections to spelling and formatting errors, and enhancements to the discussion section. These changes have been implemented to improve the overall quality and readability of the manuscript.

In conclusion, we believe that the revisions made to the manuscript have addressed the concerns raised by the reviewers and have strengthened the overall quality of the study. We are confident that the revised manuscript meets the standards for publication in PLOS ONE.

Thank you for considering our revised manuscript. We look forward to hearing from you soon.

Sincerely,

Emma Junedahl

Department of molecular and clinical medicine, Institute of Medicine University of Gothenburg, Gothenburg, Sweden
