# Supplementary material for: The evidence supporting AHA guidelines on adult cardiopulmonary resuscitation (CPR)
Source: PLoS One. 2024 Dec 17;19(12):e0309241. doi: 10.1371/journal.pone.0309241 (PMC11651587; doi:10.1371/journal.pone.0309241)
Supplement: Attachment — Submitted filename: Response to Reviewers.pdf [file pone.0309241.s004.pdf]

Re: Manuscript ID: PONE-D-24-02668

Title: "The Evidence Supporting AHA Guidelines on Adult Cardiopulmonary Resuscitation (CPR)"

Dear Editorial Team,

Thank you for the opportunity to revise our manuscript in response to the reviewers' comments. We appreciate the thorough review and constructive feedback provided by the reviewers. In this response letter, we address each of the points raised by the academic editor and reviewers.

Reviewer #1:
Reviewer #1 did not provide specific comments.

Reviewer #2:
We have addressed the reviewer's request for incorporating updated guidelines to make the article more current. Therefore, we reviewed and compared the latest formal update from 2023. Additionally, we have provided more detailed information on how recommendations were collected and reviewed in terms of LOE and COR through data processing in the R program. Specifically, we examined changes in the Class of Recommendation (COR) and/or Level of Evidence (LOE) for all the recommendations we studied in the 2020 edition. We also reviewed the two references recommended by the reviewer (Gonzalez-Del-Hoyo et al. and Fanaroff et al.) and ensured our methodology aligns with established practices. Lastly, we included an additional limitation of the study, namely that we did not include expert consensus documents, which should be considered in future research to provide a more comprehensive evaluation.

Reviewer #3:
We appreciate the reviewer's positive feedback on the insightfulness of our submission.

Conclusion:
We believe that the revisions made to the manuscript have addressed the concerns raised by the reviewers and have strengthened the overall quality of the study. We are confident that the revised manuscript meets the standards for publication in PLOS ONE.

Thank you for considering our revised manuscript. We look forward to hearing from you soon.

Sincerely,
Emma Junedahl
Research Assistant
Department of Molecular and Clinical Medicine
Institute of Medicine, University of Gothenburg
Gothenburg, Sweden
